# Wind Tunnel Measurement Systems for Unsteady Aerodynamic Forces on Bluff Bodies: Review and New Perspective

**DOI:** 10.3390/s20164633

**Published:** 2020-08-17

**Authors:** Zengshun Chen, Yemeng Xu, Hailin Huang, Kam Tim Tse

**Affiliations:** 1 School of Civil Engineering, Chongqing University, Chongqing 400045, China; xu.ym@cqu.edu.cn (Y.X.); hailinhuang@cqu.edu.cn (H.H.); 2 Department of Civil and Environmental Engineering, The Hong Kong University of Science and Technology, Clear Water Bay, Kowloon, Hong Kong, China; timkttse@ust.hk

**Keywords:** conventional wind tunnel tests, forced vibration test, hybrid aeroelastic-pressure test, bluff body aerodynamics, bluff body aeroelasticity

## Abstract

Wind tunnel tests have become one of the most effective ways to evaluate aerodynamics and aeroelasticity in bluff bodies. This paper has firstly overviewed the development of conventional wind tunnel test techniques, including high frequency base balance technique, static synchronous multi-pressure sensing system test technique and aeroelastic test, and summarized their advantages and shortcomings. Subsequently, two advanced test approaches, a forced vibration test technique and hybrid aeroelastic- force balance wind tunnel test technique have been comprehensively reviewed. Then the characteristics and calculation procedure of the conventional and advanced wind tunnel test techniques were discussed and summarized. The results indicated that the conventional wind tunnel test techniques ignored the effect of structural oscillation on the measured aerodynamics as the test model is rigid. A forced vibration test can include that effect. Unfortunately, a test model in a forced vibration test cannot respond like a structure in the real world; it only includes the effect of structural oscillation on the surrounding flow and cannot consider the feedback from the surrounding flow to the oscillation test model. A hybrid aeroelastic-pressure/force balance test technique that can observe unsteady aerodynamics of a test model during its aeroelastic oscillation completely takes the effect of structural oscillation into consideration and is, therefore, effective in evaluation of aerodynamics and aeroelasticity in bluff bodies. This paper has not only advanced our understanding for aerodynamics and aeroelasticity in bluff bodies, but also provided a new perspective for advanced wind tunnel test techniques that can be used for fundamental studies and engineering applications.

## 1. Introduction

With the development of society and economy, numerous high-rise structures have been built all over the world. For example, the heights of Burj Khalifa tower, Shanghai Tower, etc., have exceeded 600 m and the aspect ratio of the 432 Park Avenue building has achieved 15:1. Bluff bodies may experience excessive levels of vibration under the action of wind, and the effect of wind on these bluff bodies becomes more significant than the effect of seismic activities. Therefore, the prediction of wind load and response of bluff bodies is a significant consideration for a structural design engineer. Despite significant advances in computational fluid dynamics (CFDs), the wind tunnel test is still regarded as an important way to evaluate the action of wind on structures.

A high frequency base balance (HFBB) technique or a static synchronous multi-pressure sensing system (SMPSS) test technique is often carried out to obtain wind loads acting on a structure. These test techniques have been widely applied to bluff bodies to evaluate their performance. However, both the HFBB technique and the SMPSS technique are static measurements, in which wind loads are obtained from rigid test models and the effect of structural vibration (unsteady effect) is, therefore, excluded. The effect is conventionally small, but when it is in-phase with oscillating velocity the effect acts as aerodynamic damping and cannot be neglected. It has been affirmed that differences in wind loads measured from a static and a dynamic test are mainly ascribed to neglecting the unsteady effect [1]. To investigate the unsteady effect, a forced vibration wind tunnel test is usually conducted, and the test model is driven to oscillate harmonically in order to simulate the structural vibration. The results are, therefore, more accurate due to the consideration of oscillation. This technique has been used to evaluate unsteady aerodynamics in bluff bodies. The test model is forced to oscillate, and it cannot experience the actual vibration like a real structure [2]. To overcome the disadvantages in a forced vibration, an aeroelastic wind tunnel test is often performed to evaluate wind-induced vibrations of a structure; and for super-high buildings, such as Burj Khalifa Tower, it is necessary to carry out an aeroelastic test to evaluate its aerodynamics and aeroelasticity. The aeroelastic technique can well consider the effect of structural vibration, but only the aeroelastic response is obtained and the wind load cannot be observed simultaneously. Meanwhile, the aerodynamic damping identified from an aeroelastic response using a proper mathematical technique (i.e., random decrement technique) may not be accurate [3].

The aforementioned wind tunnel test techniques have their advantages and disadvantages in evaluating aerodynamics and aeroelasticity in bluff bodies. Few studies have comprehensively overviewed the characteristics and developments of those wind tunnel test techniques in recent years and reviews for advanced wind tunnel tests like hybrid aeroelastic-pressure/force balance wind tunnel test techniques are very limited. In this paper, the conventional wind tunnel test techniques, including the HFBB test, SMPSS test and aeroelastic wind tunnel test, are reviewed in detail. Then two advanced wind tunnel test techniques, a forced vibration test technique and a hybrid aeroelastic- force balance (HAFB) test technique, are comprehensively overviewed in light of the limitation of conventional wind tunnel test techniques. The characteristics of unsteady forces measured from the conventional and advanced wind tunnel tests are presented. Finally, new perspectives for advanced wind tunnel test techniques are summarized.

## 2. Conventional Wind Tunnel Test Techniques

### 2.1. HFBB Test

The high frequency base balance (HFBB) test technique was proposed in the early 1980s [4,5] and has been developed as an economical and effective alternative approach to the more involved aeroelastic test. It is widely used for the prediction of the wind-induced base shear and moment of high-rise structures [6,7,8,9]. In the HFBB wind tunnel test technique, the base forces and bending moments in different directions are observed through the balance equipped at the bottom of the model. The main apparatus used in the HFBB test is the force/bending moment sensor system. The system can be used to measure forces and bending moments in three directions acting on the test model. All data are obtained from the system at an appropriate frequency, for example, 500 Hz. With the time history of forces and bending moments, spectra of generalized force and response can be obtained through the Fast Fourier Transform (FFT). As a result, characteristics of aerodynamic forces of a bluff body can be analyzed.

The technique involves the base force measurements of a nearly rigid scale model, which models only the external geometry of a structure. If the model is not rigid enough and responds dynamically to the wind force, the interaction between the dynamic response and the aerodynamic force would contaminate the base force measurements [10]. Therefore, the test model is made as rigid as possible. In addition, the technique was mainly developed for uncoupled linear mode shapes, as the coupled nonlinear mode shape may lead to complicated issues [8,9]. It has been affirmed that the dynamic response can be sufficiently evaluated through the first three fundamental mode shapes [11] in two sway directions and one twist direction. Each mode shape is hypothesized as linear translational mode shape. However, in practice, the contribution of higher modes to the response calculation may be considerable and in this case mode shape correction factors are introduced to consider the contribution. The factors have been empirically suggested in many studies [8,12].

In brief, the HFBB test technique is an effective and expeditious way to obtain exact overall wind loads (i.e., base shear force and base overturning moment). Furthermore, only one test is needed to determine wind-induced responses for a series of structures with the same geometry. However, this technique has some limitations: The ideal linear translational mode shape and constant torsional mode shape of a HFBB are assumed or a correction factor is needed; only the fundamental modes of the two translational and one torsional mode shapes are concerned in the response analysis, and the higher mode shapes are neglected; the test model should be rigid enough and no significant motions during the test; the local wind force characteristics cannot be obtained.

There have been lots of investigations on conventional buildings through the HFBB test and the technique has been developed for several decades [13,14,15]. In recent years, with the development of irregular buildings, some researchers have focused on the characteristics of aerodynamic forces on unconventional structures (twisted building, tapered building) [16,17]. In addition, some new analysis methods based on the HFBB test have been proposed in order to address the disadvantage of unreal mode shape [13,18]. However, the inherent limitation that the HFBB test cannot measure the distributed pressure still exists. Furthermore, the model is static and the unsteady effect is, therefore, excluded.

### 2.2. SMPSS Test

A SMPSS wind tunnel test technique is used to observe the wind pressure on the surface of the test model synchronously through the synchronous multi-pressure sensing system. Different from the HFBB test technique, pressure taps are installed at different levels of the test model and the distributed pressure is therefore recorded using a high-speed scanning pressure equipment. The base shear force and moment of the test model can be evaluated by using the high frequency pressure integration (HFPI) method [19,20]. As pointed out in previous studies [21,22], the SMPSS test can provide the same output of the overall wind forces as that in a HFBB test if the pressure taps are installed at a fine resolution over the surfaces of a test model. Wind-induced vibrations of a test model can therefore be evaluated by the integrated overall wind force. It is noteworthy that the contribution of higher modes to the response can be estimated as the distributed force at different levels is obtained. Furthermore, the local aerodynamic characteristics of a test model can also be analyzed [23,24]. In addition, a SMPSS test with a lumped mass test model allows to estimate the contribution of both the inertial force and the external pressure to the shear, torque, bending moment along the height of the test model.

A test model in a SMPSS test should be rigid enough to avoid evident wind-induced vibration so that the pressure measured in the test is precise [25]. Furthermore, in order to reduce the impact of pressure tap tubes on the signal, the length of the tube is usually less than 1.4 m.

The SMPSS technique has been widely used for estimating wind loads and wind-induced vibrations of structures due to the above illustrated advantages. However, there are some technical drawbacks inherent to it. For instance, a SMPSS test may make use of a large number of pressure taps to accurately predict wind forces acting on a test model. Furthermore, it is difficult and prohibitive to install the pressure taps especially in the presence of complicated geometric forms (i.e., buildings with balconies or screens covered by the facade). For bluff bodies, the static measurements are prone to be affected by model motion. Lastly, the SMPSS test cannot consider the unsteady effect and the influence of structural vibration is excluded.

### 2.3. Aeroelastic Test

An aeroelastic test technique makes use of an elaborate test model to directly obtain the aeroelastic process. The model is designed to not only have the exterior geometry of the prototype structure, but also the relevant dynamic parameters such as stiffness, mass and effective damping [22], so that the test model responds under the action of wind in the same way as the prototype would. Apart from the aforementioned parameters, the flow environment in a wind tunnel should also be well considered. However, it is hard to guarantee the exact scale ratio between the test model in a wind tunnel and that in the real world. High Reynolds number flow is usually assumed for high-rise structures, indicating that the flow is regarded as laminar flow and the viscous forces of the fluid are therefore neglected. For the case that the action of wind on a structure is sensitive to Reynolds number (i.e., wind effect on circular cylinders), the Reynolds number should be well considered.

**Figure 1:**
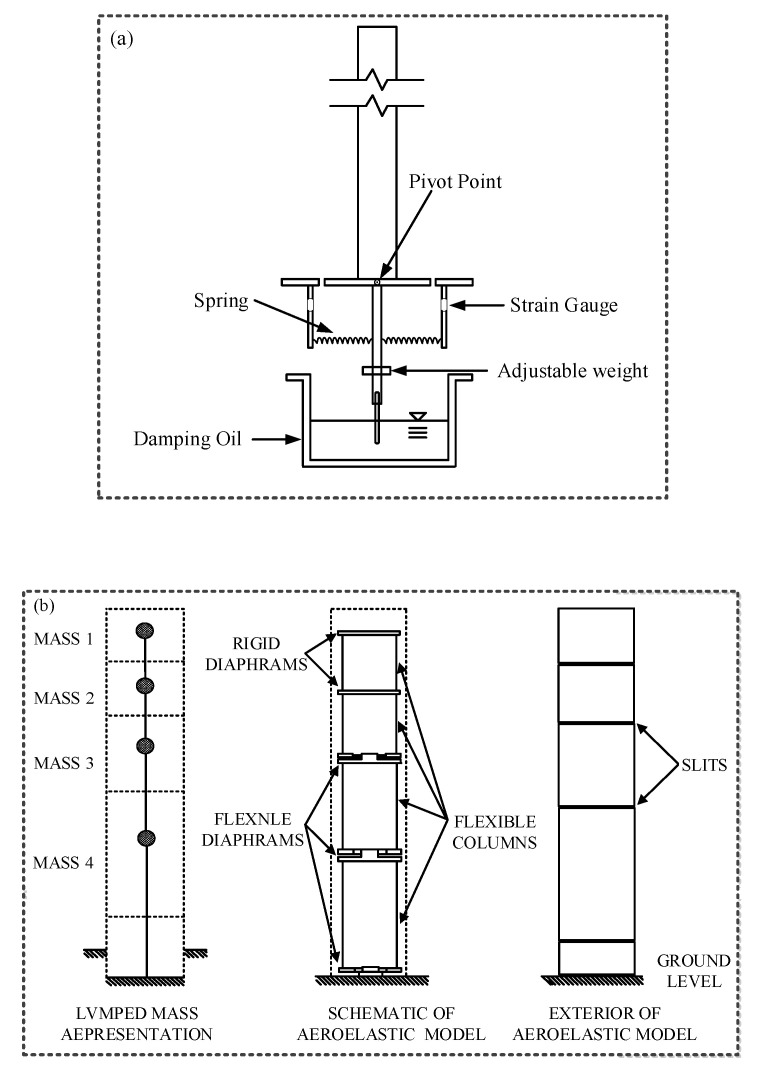
Typical schematic aeroelastic models: (**a**) A stick model; (**b**) a multi-degree-of-freedom model, after [26].

Typical schematic aeroelastic models are presented in Figure 1. In Figure 1a, the damping ratio of a test model is adjusted by a plate which is immersed into an oil tank. The mass and fundamental frequency are adjusted by changing the position of the adjustable weight and the spring, respectively. The test model is pivoted to oscillate in one sway direction under the action of wind and therefore only linear mode shape is considered. This kind of test models is the so called “stick model”. For high-rise structures, it is sufficient to consider the first two fundamental mode shapes and assume the mode shape as linear mode shape. For particular bluff bodies, higher mode shapes are important and in this case, another kind of aeroelastic models, which consists of several lumped elements is adopted [26]. An example of a lumped element model is shown in Figure 1b.

Through an aeroelastic test, (1) wind-induced oscillations of a structure can be directly measured; (2) fluid-structure interactions can be well included; (3) high mode shapes of a test model can be well considered. Therefore, this test technique is widely accepted and used for evaluating wind-induced vibrations of structures. Meanwhile, the disadvantages of an aeroelastic test should also be highlighted: (1) The design and construction of an aeroelastic model is time-consuming; (2) during the test, dynamic parameters may vary with oscillating amplitude; (3) the wind loading information, which is required by engineers, cannot be obtained.

In summary, by using a HFBB or a SMPSS wind tunnel test technique, static base forces or pressures of a test model are observed, which can be used for response predictions. The main problem of the test techniques is that the “unsteady” effect that has been proved to have a significant effect on response predictions is neglected. By using an aeroelastic test technique, wind-induced vibrations of a test model, which contains the “unsteady” effect, can be directly observed. However, wind forces of the test model which are required by the engineers cannot be obtained. Additionally, the aeroelastic test is complicated and time-consuming. To overcome the shortcomings in the conventional wind tunnel test techniques, advanced wind tunnel test techniques (e.g., forced vibration test techniques and hybrid aeroelastic-pressure/force test techniques) that can include the unsteady effect are required.

## 3. Advanced Wind Tunnel Test Techniques

### 3.1. Forced Vibration Test Technique

**Figure 2:**
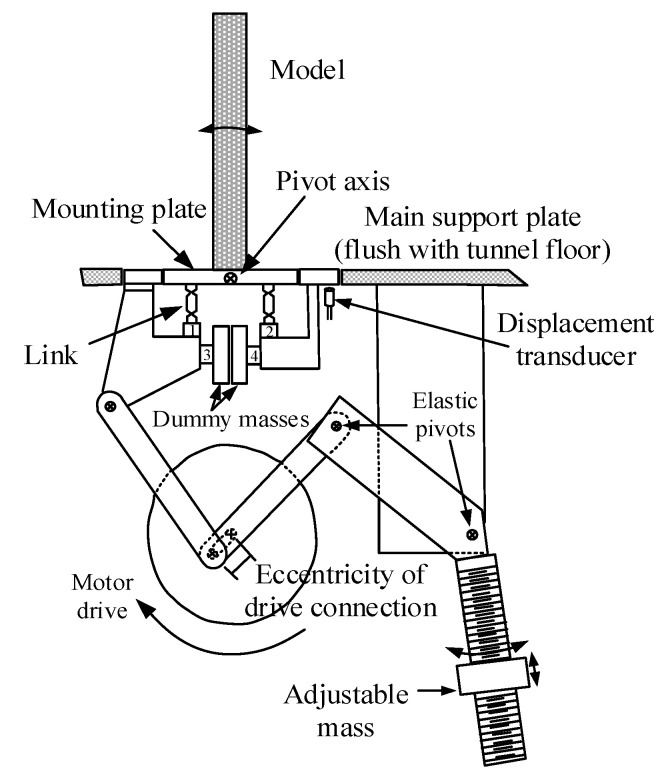
A forced vibration test with respect to base force measurement [27].

Due to the different limitations of static measurement tests (HFBB and SMPSS) and aeroelastic test, a forced vibration test is often carried out to evaluate unsteady wind forces of structures. A forced vibration test system mainly includes a test model, an actuator device, a signal generator, a power amplifier, a force measurement system and a response measurement system. An example of forced vibration test systems is given in Figure 2. The test model was mounted on a plate through two passive elastic pivots and is driven to oscillate harmonically in the crosswind direction by using the driving motor system. The test model was rigid enough and only the sway linear mode shape was considered. The base forces under different oscillating amplitudes and reduced wind velocities were measured from load cells installed underneath the mounting plate.

**Figure 3:**
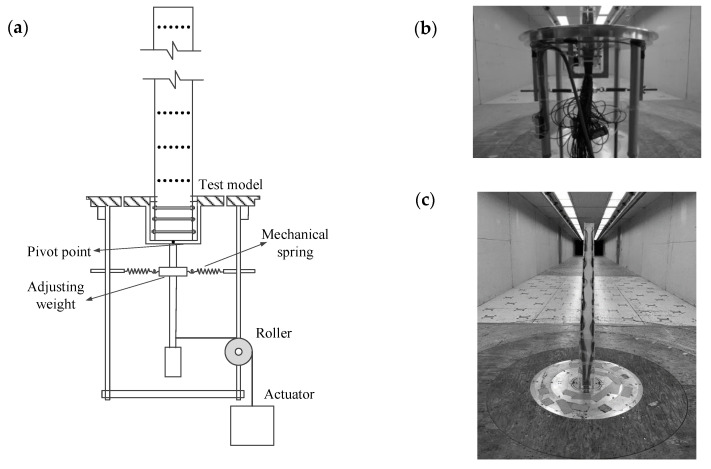
Schematic diagram of a forced vibration device: (**a**) a forced vibration test rig; (**b**) a forced vibration model in a wind tunnel; (**c**) the global view of the forced vibration model in a wind tunnel.

As mentioned before, unlike distributed pressures, base forces do not include local wind force characteristics. Bearman and Currie [28] and Bearman and Obasaju [29] performed a forced vibration test of a two-dimensional cylinder with respect to unsteady pressure measurements. The characteristics of unsteady pressures were analyzed. Cooper et al. [30] carried out a forced vibration test of a three-dimensional tapered prism with respect to unsteady pressure measurements. The effect of oscillating amplitude and wind velocity on the unsteady pressure was discussed. Most recently, Banfi and Carassale [31] and Li et al. [32] devised forced vibration systems for two-dimensional cylinders. Chen et al. [1,33] have further investigated the aerodynamic damping of vertical and inclined prisms through a forced vibration test. The response and wind pressure of the model were measured synchronously. It has been confirmed that the accuracy of unsteady aerodynamic forces can be significantly improved ascribed to the consideration of structural vibration. The schematic diagram of the forced vibration device is in Figure 3.

The above illustration indicates that a forced vibration test model with pressure measurements includes much more information than that with respect to base force measurements. Furthermore, by integrating unsteady distributed pressures along the height of a prism, observed from a forced vibration wind tunnel test, unsteady wind forces of the prism can be estimated. Due to the advantages of a forced vibration technique, it has been widely used for flutter derivative identification [34,35], aerodynamic force and damping analysis and spanwise correlation analysis [32,36]. However, a test model in a forced vibration wind tunnel test technique oscillates harmonically, which cannot experience the same wind forces as a model that is able to respond as a real structure would. That is to say, only one-way coupling effect is included.

### 3.2. Hybrid Aeroelastic-Force Balance Test Technique

**Figure 4:**
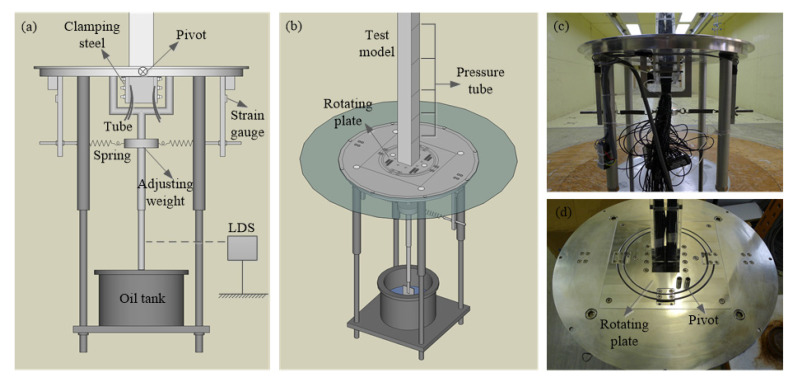
Hybrid aeroelastic-force balance (HAFB) system: (**a**) Plan view of the test rig; (**b**) stereogram of the test rig; (**c**) test rig in a wind tunnel; (**d**) details of rotating plate and pivot [25].

However, the forced vibration test technique only takes parts of unsteady effect into account, the results of the test were not the same as the results of the real structure. The hybrid aeroelastic-force balance (HAFB) test technique can reflect the real results including a fully unsteady effect. In a HAFB technique, a test model oscillates freely under the action of wind and meanwhile unsteady wind forces of the test model are sampled. As the test model responds as a real structure and the fluid-structure interaction is well simulated, the observed unsteady wind forces are more accurate than those observed from a forced vibration test. The HAFB test technique was composed of two parts, including free vibration measurement and unsteady pressure measurement. In this section, the mechanism of the test technique was clarified clearly and the development of the HAFB test technique was introduced. An example of HAFB test system is given in Figure 4. The tip response of the test model was determined by the response observed from the LDS through a proper calibration (obtain the relationship between the tip response and the LDS observed response). The oscillating frequency $f_{s}$ and mechanical damping ratio $\xi_{s}$ of the HAFB were determined by a signal of free decay response.

Besides, the pressure measurement was synchronous with the response measurement, and the synchronization was realized by inputting a random and periodical signal generated by a signal generator. The pressure was sampled by using a synchronous multi-pressure sensing system (SMPSS). The sampling frequency and the duration time of unsteady pressure measurement are the same as those of the free vibration measurement.

**Figure 5:**
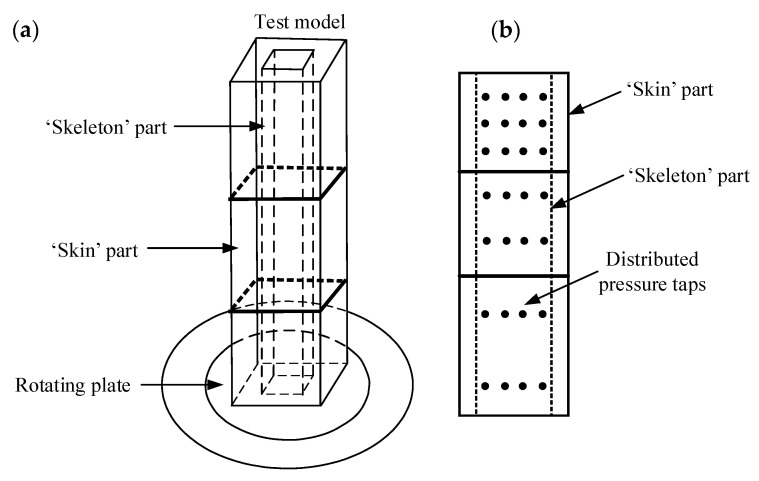
A base force–pressure–aeroelastic integrated model, after [37]: (**a**) Global view of the hybrid aeroelastic-force-pressure measurement system; (**b**) Side view of the bluff body in hybrid aeroelastic-force-pressure measurement system.

Very few studies have focused on the HAFB technique. Chiara Pozzuoli [37] has developed a hybrid aeroelastic-force-pressure measurement system. Distributed pressures, overall forces, acceleration and displacements of a test model were simultaneously obtained by using the system (Figure 5).

**Figure 6:**
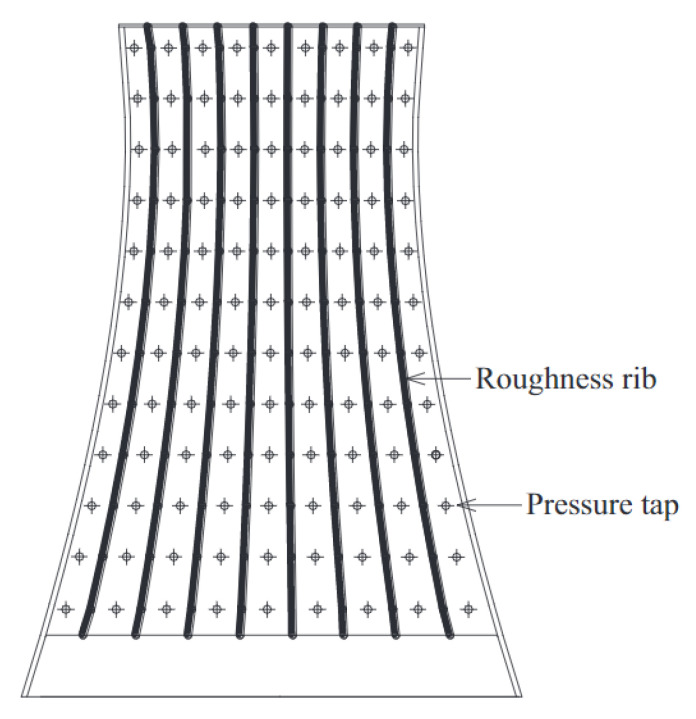
A HAFB model of a cooling tower [39].

The test model in Figure 5 is a continuous “skin–skeleton” type aeroelastic model that consists of an external non-structural “skin” part and an inner structural “skeleton” part. The inner three boxes are rigid and connected at three different levels. The test results are used for analyzing characteristics of unsteady crosswind forces and identifying the aerodynamic damping of the test model. However, the experimental activity in the boundary layer wind tunnel was a laborious stage of work and inconvenient in practical use. It is not necessary to set up a “skin–skeleton” type model and a stick is enough to investigate aerodynamic and aeroelastic characteristics of a tall building. Furthermore, the test model cannot experience large oscillations under the action of wind, suggesting galloping-like self-excited vibrations cannot occur. Another example of a HAFB system has been devised to estimate wind loads and wind-induced vibrations of a cooling tower by using a modified equivalent beam-net design method [38,39,40], as shown in Figure 6. The test model can simulate high mode shapes of a cooling tower. However, as mentioned by the authors, there exist considerable differences (achieved to 10%) between the test model and the real cooling tower.

**Figure 7:**
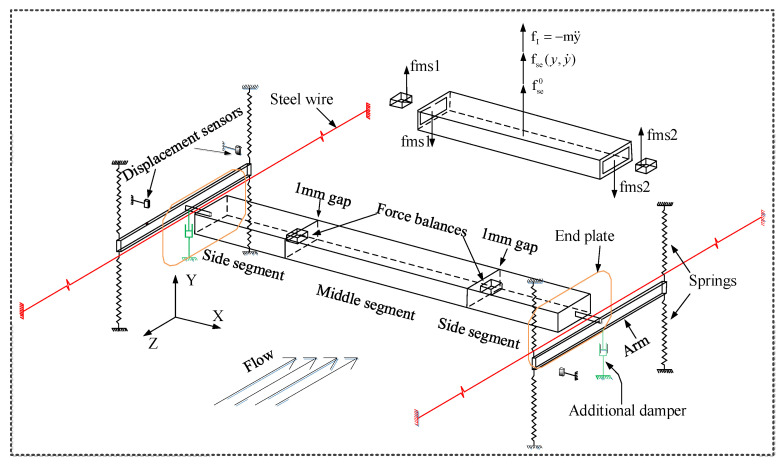
A spring-suspended system that is used for based force and response measurements [41,44].

Most recently, Gao and Zhu [41,42] devised a spring-suspended system (Figure 7) that can observe unsteady base forces and aeroelastic vibrations of a two-dimensional test model. The stiffness and damping of the system were adjusted by changing springs and additional dampers that were installed at the two ends of the system. The system was a weakly nonlinear system and the amplitude-dependent nonlinear mechanical frequency and damping were identified before the test. The unsteady base force and response of the test model were measured by force balances and displacement sensors, respectively. It is noteworthy that galloping-like large amplitude oscillations of the test model occur. Correspondingly, unsteady self-excited forces during galloping were observed. The test system has been proved to be reliable and accurate to obtain the unsteady self-excited force of the test model. However, it is base force measurement and characteristics of local unsteady wind forces (i.e., span-wise correlation) cannot be analyzed. Furthermore, the system was used for two-dimensional deck sections and cannot be used for three-dimensional prisms. Besides, Chen et al. [25,43] developed a mathematical model to quantify the unsteady self-excited forces acting on a slender by HAFB wind tunnel tests, and found that the forces obtained from the tests can be used to predict the galloping response.

## 4. Unsteady Aerodynamic Forces Measured from Advanced Wind Tunnel Test Techniques

To explain the effectiveness of the advanced wind tunnel test techniques, unsteady aerodynamics (e.g., aerodynamic forces and aerodynamic damping) and aeroelasticity (e.g., galloping) measured from the techniques are reviewed.

### 4.1. Characteristics of Unsteady Wind Force

**Figure 8:**
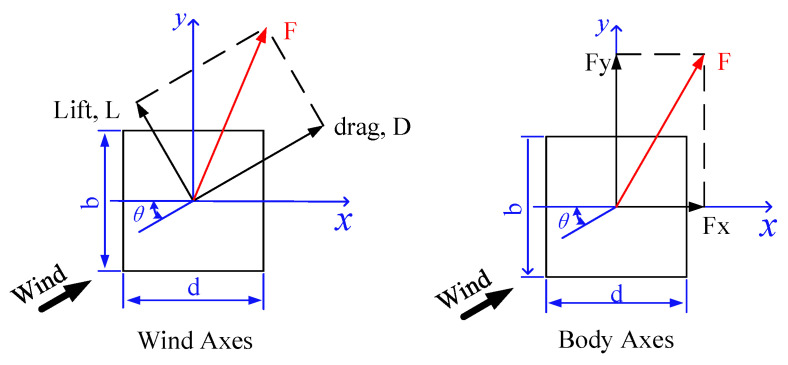
Wind force components of a square prism [37]

The distributed pressure as well as the overall wind load of a prism can be evaluated from a SMPSS test. Wind pressures on the windward face of a structure are positive as the approaching flow directly acts on the face whereas they are negative on the other three faces (leeward face and side faces) due to suction. Wind forces in the along wind direction are defined as drag force and those in the crosswind direction are defined as lift force (Figure 8).

For three-dimensional prisms, the local and overall mean, root mean square (RMS) lift and drag force coefficient are defined as follows. It should be noted in the crosswind direction the mean lift force coefficient is close to zero and is therefore neglected. The base moment coefficient can also be evaluated from the observed local pressure coefficients.

The local wind force coefficients of a test model at height *z* above the ground are defined as

(1) $${\;\bar{C}}_{D}\left(z\right)=\frac{F_{D}\left(z\right)}{A_{z}q_{H}},{\;\tilde{C}}_{D}\left(z\right)=\frac{\sigma_{F_{D}\left(z\right)}}{A_{z}q_{H}},{\;\tilde{C}}_{L}\left(z\right)=\frac{\sigma_{F_{L}\left(z\right)}}{A_{z}q_{H}}$$

where ${\;\bar{C}}_{D}\left(z\right),{\;\tilde{C}}_{D}\left(z\right)$ and ${\tilde{C}}_{L}\left(z\right)$ are the local mean drag force coefficient, the local RMS drag force coefficient and the local RMS lift force coefficient, respectively. $F_{D}\left(z\right),\sigma_{F_{D}\left(z\right)}{\;\mathrm{and}\;\sigma }_{F_{L}\left(z\right)}$ are the mean drag force, the RMS drag force and the RMS lift force, respectively. $A_{z}$ is the sectional area. $q_{H}=\frac{1}{2}\rho U^{2}$, where ρ is air density and U is wind velocity.

Integrating the obtained local wind force coefficients at each level, the generalized force coefficients are expressed as

(2) $${\;\bar{C}}_{D}=\frac{\int_{0}^{H}{\;\bar{C}}_{D}\left(z\right)\cdot A_{z}\cdot \phi \left(z\right)\mathrm{dz}}{\mathrm{DH}},{\;\tilde{C}}_{D}=\frac{\int_{0}^{H}{\;\tilde{C}}_{D}\left(z\right)\cdot A_{z}\cdot \phi \left(z\right)\mathrm{dz}}{\mathrm{DH}},{\;\tilde{C}}_{L}=\frac{\int_{0}^{H}{\;\tilde{C}}_{L}\left(z\right)\cdot A_{z}\cdot \phi \left(z\right)\mathrm{dz}}{\mathrm{DH}},$$

where, ${\;\bar{C}}_{D},\;{\;\bar{C}}_{D}$ and ${\tilde{C}}_{L}$ are the generalized mean drag force coefficient, the RMS drag force coefficient and the RMS lift force coefficient, respectively. D and H are the width and height; ϕ(z) denotes the mode.

The base force coefficient is expressed as

(3) $${\bar{C}}_{\mathrm{MD}}=\frac{{\;\bar{F}}_{\mathrm{MD}}}{{\mathrm{DH}}^{2}q_{H}},{\;\tilde{C}}_{\mathrm{MD}}=\frac{{\;\tilde{F}}_{\mathrm{MD}}}{{\mathrm{DH}}^{2}q_{H}},{\;\bar{C}}_{\mathrm{ML}}=\frac{{\;\bar{F}}_{\mathrm{ML}}}{{\mathrm{DH}}^{2}q_{H}},{\;\tilde{C}}_{\mathrm{ML}}=\frac{{\;\tilde{F}}_{\mathrm{ML}}}{{\mathrm{DH}}^{2}q_{H}}$$

where ${\;\bar{C}}_{\mathrm{MD}},{\;\tilde{C}}_{\mathrm{MD}},{\;\bar{C}}_{\mathrm{ML}}{\;\mathrm{and}\;\tilde{C}}_{\mathrm{ML}}$ denote the mean drag base moment, the RMS drag base moment, the mean lift base moment and the RMS lift base moment, respectively; ${\bar{F}}_{\mathrm{MD}},{\;\tilde{F}}_{\mathrm{MD}},{\;\bar{F}}_{\mathrm{ML}}{\;\mathrm{and}\;\tilde{F}}_{\mathrm{ML}}$ denote the mean drag force, the RMS drag force, the mean lift force and the RMS lift force, respectively.

Based on observed wind pressures and the above definitions, static wind force characteristics of structures have been comprehensively analyzed [9,21,45,46]. Most recently, static wind force characteristics of backward or forward inclined prisms were investigated [47]. The effect of inclination on the force coefficients was analyzed. It was pointed out that the base shear force and moment tend to decrease with increasing the inclinations apart from a small backward inclination case. The possible reasons were illustrated in terms of base force spectra, pressure coherences of the side face pressures and vortex shedding frequencies.

**Figure 9:**
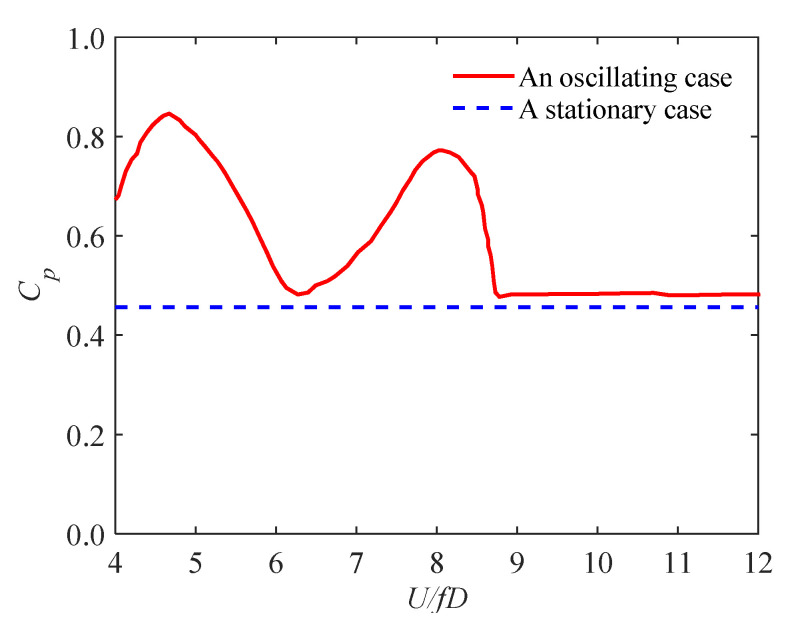
Fluctuating pressure measured on a side face, after [29].

**Figure 10:**
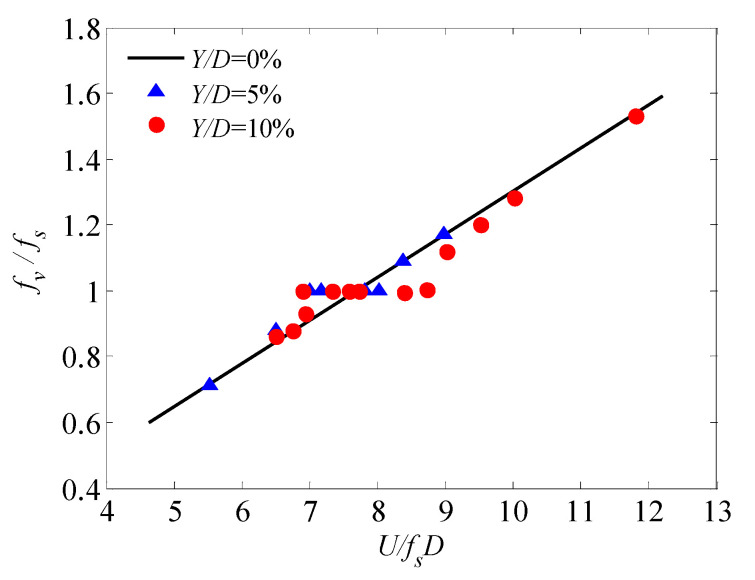
Lock-in phenomenon, after [29].

Many studies have focused on unsteady wind forces on bluff bodies. Bearman and Obasaju [29] have investigated the pressure fluctuation of oscillating two-dimensional circular and square-sectional cylinders. Both the mean and fluctuating crosswind pressures of the cylinders at or away from the lock-in range were observed, and substantial differences between the two cylinders were discovered (Figure 9). It was found that, at high wind speeds, oscillating pressures are in close agreement with that measured from a static test model, suggesting that the pressures are under quasi-steady state and the effect of structural oscillation is slight. At low wind speeds (around the lock-in range), a significant peak took place (Figure 10), which was ascribed to the interaction of structural motion and vortex shedding. The vortex shedding process will impart forces on prisms and the prisms may respond to these forces. When the shedding frequency is equal or in close agreement to the natural frequency of the prism, a coupling between the response and the wake exists. In this case, the oscillating frequency of the prism dominates the vortex shedding frequency of wind flow at a certain wind speed range and the range increases as the structural damping decreases.

In steady flow, bluff bodies can undergo vortex-induced-vibrations (VIVs) because of the von Karman vortices generated in wake flow which will create unbalanced forces on the structure. VIVs of prisms occur at the lock-in range where the exciting frequency may close to the natural frequency of the body. In addition, downstream structures behind the bluff bodies generating vortices may also undergo VIV because of the impingement of induced flow and the low pressure core region of the vortices [48]. Because of the practical and theoretical importance of VIV, it has received considerable attentions [48,49,50,51,52,53]. The phenomena as well as the mechanism about the VIV of bluff bodies with simple configurations were primarily investigated. The commonly accepted interpretation of the mechanism underlying the VIV is that there is a net flux energy from the fluid flow into a structure and, with respect to the structure, negative damping is set up which reduce the total damping of the structure. In the case of low structural damping and mass of a structure, oscillations of the body may be enlarged. It is usually regarded as the lower the structural damping and mass are, the greater the VIV of the body are. However, the VIV limits themselves to amplitudes on the order of the body dimension even for the low structural damping and mass case. This feedback mechanism is implicitly assumed to be nonlinear. The flow and thereby the negative damping are altered by the body motion. The altered flow provides less net energy flux to the structure at some oscillating amplitudes and equilibrium of energies is achieved. The oscillating frequency not always coincides with the natural frequency of the structure. Oscillations may occur at twice or three times the shedding frequency, which may be ascribed to the effect of the airflow added mass and the geometry of the vortex shedding street [54,55].

**Figure 11:**
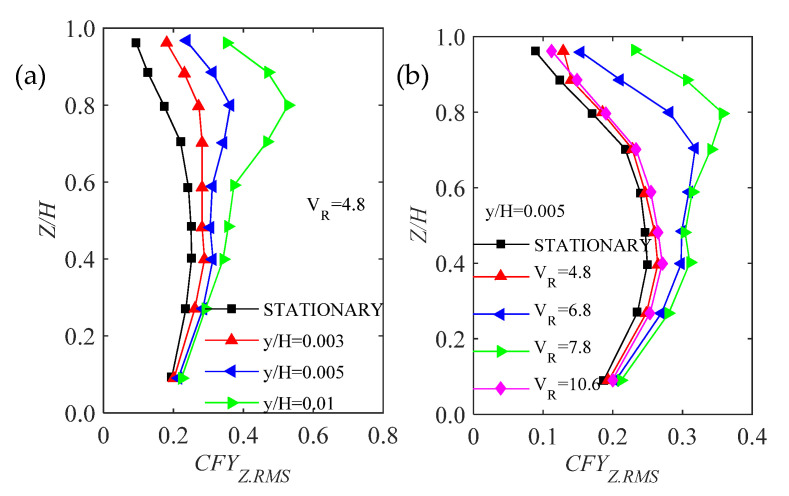
The effect of oscillating amplitude and reduced velocity on the sectional crosswind force coefficients, after [30]: (**a**) with respect to tip amplitude of the bluff body; (**b**) with respect to the reduced wind speed.

Cooper et al. [30] and Katagiti et al. [56,57] investigated unsteady pressures of bluff bodies. The effect of oscillating amplitude and reduced velocity on the sectional alongwind and crosswind force coefficients were analyzed. It was found that, in the alongwind direction, oscillating amplitude and wind velocity have a slight effect on the observed force coefficients, whereas they have a significant effect on the observed force coefficients in the crosswind direction. An example, local force coefficients in the crosswind direction, is given in Figure 11. It shows that, at low wind speeds, the local force coefficients increase with oscillating amplitude whereas at high wind speeds, the local force coefficients are in close agreement with that of a stationary case.

**Figure 12:**
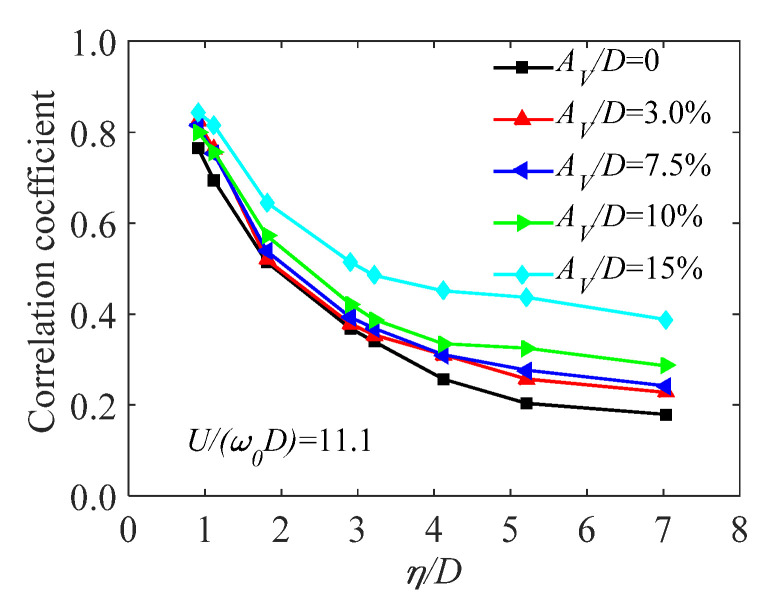
Effect of the vertical amplitude on the spanwise correlation of aerodynamic forces acting on an oscillatory cylinder around the vortex lock-in range [32].

Based on observed unsteady pressures, spanwise correlations of cylinders have been analyzed [32,58]. It is noteworthy that, at the vortex lock-in range, the spanwise correlation increases considerably with oscillating amplitude and away from the vortex lock-in range, the spanwise correlation changes slightly with increasing oscillating amplitude, suggesting that oscillating amplitude has a significant effect on spanwise correlation around the vortex lock-in range and has a slight effect on spanwise correlation away from the vortex lock-in range. A study [32] also pointed out that the vibrating pattern, wind incidence and Reynolds number also have considerable effects on spanwise correlation and the effects cannot be neglected (Figure 12).

The above studies have not only advanced our understanding of characteristics of unsteady wind forces of structures, but also provide a foundation for further analysis. The following section will introduce the identified aerodynamic damping based on the unsteady wind forces obtained from a forced vibration test.

### 4.2. Aerodynamic Damping Force

The observed unsteady wind force is composed of a random component due to unsteady wake effects and turbulence, and a motion-induced force component. Based on a previous study [10], it is expressed as

(4) $$W\left(t\right)=W_{l}\left(t\right)+W_{m}\left(t\right)=\frac{1}{2}{\rho U}^{2}{\mathrm{DH}}^{2}\left(C_{l}\left(t\right)+C_{m}\left(t\right)\right)$$

where W(t) is the observed unsteady wind moment; $W_{l}\left(t\right)$ and $W_{m}\left(t\right)$ are the random wind moment component and the motion-induced moment component, respectively; $C_{l}\left(t\right)$ and $W_{m}\left(t\right)$ are the random wind force coefficient and the motion-induced force coefficient, respectively.

The corresponding base force is written as

(5) $$F\left(t\right)=F_{l}\left(t\right)+F_{m}\left(t\right)=\frac{1}{2}{\rho U}^{2}\mathrm{DH}\left(C_{l}\left(t\right)+C_{m}\left(t\right)\right)$$

where F(t) is the observed unsteady wind force; $F_{l}\left(t\right)$ and $F_{m}\left(t\right)$ are the random wind force component and the motion-induced force component, respectively.

A complex aerodynamic impedance, $K_{a}$ is defined and expressed as

(6) $$K_{a}y=-\frac{1}{2}{\;\rho U}^{2}{\mathrm{DHC}}_{m}$$

Then, a dimensionless form of impedance $G_{a}$ is defined as

(7) $$G_{a}=\frac{K_{a}}{2\omega^{2}M_{s}\eta }$$

where $M_{s}\eta$ denotes the generalized mass of the prism. For a test model with linear mode shape, it is expressed as

(8) $$M_{s}\eta =\frac{{\rho \mathrm{HD}}^{2}}{3}$$

As mentioned, the motion-induced force can be divided into an aerodynamic stiffness term and an aerodynamic damping term ($G_{a}=\lambda +\mathrm{iu}$). The real part λ corresponds to the aerodynamic stiffness term and the imaginary part u corresponds to the aerodynamic damping term. Then, the base moment is re-written expressed as

(9) $$W\left(t\right)=\frac{1}{2}{\rho U}^{2}{\mathrm{DH}}^{2}\left(C_{l}\left(t\right)-\frac{4}{3}D\left({\lambda \omega }^{2}y+\frac{\mu }{\omega }\;\dot{y}\right)\right)$$

In a forced vibration test, the test model oscillates harmonically, and the tip response can be expressed as

(10) $$y\left(t\right)=\;\hat{y}\cos\left(2\pi \mathrm{ft}\right)$$

where y(t) is the tip displacement response and $\hat{y}$ is tip oscillating amplitude.

Considering the orthogonality of trigonometric functions, yields,

(11) $$I_{1}=\underset{T\to \infty }{\lim}\frac{1}{T}\int_{0}^{T}W\left(t\right)\cos\left(\omega t\right)\mathrm{dt}$$

(12) $$I_{2}=\underset{T\to \infty }{\lim}\frac{1}{T}\int_{0}^{T}W\left(t\right)\sin\left(\omega t\right)\mathrm{dt}$$

Then, the aerodynamic stiffness and damping coefficients are expressed as

(13) $$\lambda =-\frac{3}{\rho {\left(2\pi f\right)}^{2}D^{2}H^{2}\;\hat{y}}I_{1}$$

(14) $$\mu =\frac{3}{\rho {\left(2\pi f\right)}^{2}D^{2}H^{2}\;\hat{y}}I_{2}$$

**Figure 13:**
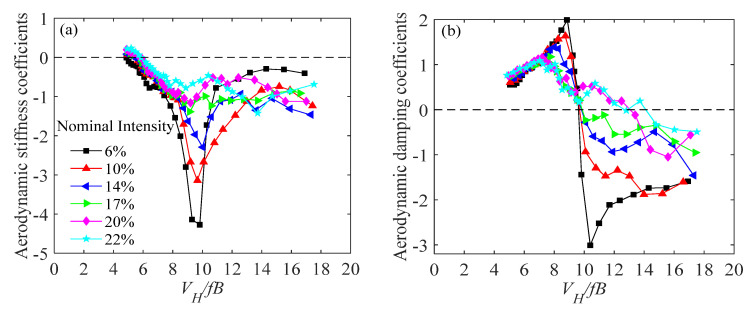
Aerodynamic stiffness and damping coefficients of a prism (**a**) aerodynamic stiffness coefficients; (**b**) aerodynamic damping coefficients, after [10].

It should be clarified that $I_{1}$ and $I_{2}$ can be derived from the time-history base force or the time-history pressure measured from a forced vibration test. By using the identification scheme introduced above, Steckley [10] identified the aerodynamic stiffness and aerodynamic damping coefficients of bluff bodies. The aerodynamic stiffness and damping coefficients of the prisms under different oscillation amplitudes and turbulence intensities observed in a previous study are presented in Figure 13. Figure 13a shows that the aerodynamic stiffness coefficients are negative under all reduced velocities. Figure 13b shows that the aerodynamic damping coefficients are positive at low wind speeds and are negative at high wind speeds. The peaks of the aerodynamic stiffness coefficients occur at the reduced wind speed around 10 which is in the vortex lock-in range. In this range, the aerodynamic damping coefficients change from positive to negative. Furthermore, the oscillating amplitude has a great effect on the aerodynamic stiffness and damping coefficients. The magnitudes of the aerodynamic stiffness and damping tend to increase with oscillating amplitude. In addition, turbulence intensity also has an impact on the two terms. The magnitudes tend to decrease with increasing oscillating amplitude. The effect of aspect ratio has also been investigated and it was found that the aerodynamic damping and stiffness coefficients are not sensitive to the aspect ratio apart from the aspect ratio of 6.67.

With linear mode shape assumption, based on random vibration theory, the variance of the tip response of a test model is estimated by

(15) $${\;\bar{y}}^{2}=\frac{1}{K_{s}^{2}}\int_{0}^{\infty }{\left|H\left(f\right)\right|}^{2}S_{\mathrm{FF}}\left(f\right)\mathrm{df}$$

where $\;\bar{y}$ is the variance of the tip response; $K_{s}$ is the generalized stiffness; f is the frequency; $S_{\mathrm{FF}}\left(f\right)$ is the spectrum of generalized force that is obtained from a HFBB test; H(f) is the modulus of the mechanical admittance function and is expressed as

(16) $$H\left(f\right)=\frac{1}{\left(1-{\left(f/f_{s}\right)}^{2}+i2\xi_{s}\left(f/f_{s}\right)\right)}$$

where $f_{s}$ is the natural frequency; $\xi_{s}$ is the structural damping ratio.

It should be emphasized that, for high-rise prisms and buildings, the aerodynamic stiffness force component is small and is often neglected, and only the aerodynamic damping force component is concerned [59]. The above analytical scheme has been proved to be reasonable and reliable in aerodynamic damping identification. Following the analytical scheme, a few studies have identified the aerodynamic damping of several kinds of prisms [30,56,60]. Substituting the identified results into Equation (16), the mechanical admittance is re-written as

(17) $$H\left(f\right)=\frac{1}{\left(1-{\left(f/f_{s}\right)}^{2}+i2\left(\xi_{s}+\xi_{a}\right)\left(f/f_{s}\right)\right)}$$

Substituting Equation (16) into (15), the response predictions can be improved.

For the purpose of convenient use, nonlinear mathematical models of the identified aerodynamic damping have been developed. Watanabe et al. [61] have proposed an empirical mathematical function which is a function of oscillating amplitude and reduced wind velocity, to model the aerodynamic damping. The empirical model is expressed as

(18) $$F_{d}=-F_{1}\sin\left(\chi \right)+F_{2}\cos\left(\chi \right)+F_{p}$$

(19) $$F_{1}=\frac{-2H_{s}\left(U/U_{\mathrm{cr}}\right)A_{p}}{{\left(1-{\left(U/U_{\mathrm{cr}}\right)}^{2}\right)}^{2}+4H_{s}{\left(U/U_{\mathrm{cr}}\right)}^{2}}$$

(20) $$F_{2}=\frac{\left(U/U_{\mathrm{cr}}\right)\left(1-{\left(U/U_{\mathrm{cr}}\right)}^{2}\right)A_{p}}{{\left(1-{\left(U/U_{\mathrm{cr}}\right)}^{2}\right)}^{2}+4H_{s}{\left(U/U_{\mathrm{cr}}\right)}^{2}}$$

where $F_{d}$ is aerodynamic damping coefficients; $U_{\mathrm{cr}}$ is the vortex wind speed; χ, $H_{s}$ and $A_{p}$ are parameters of the function, which are functions of oscillating amplitude.

Chen [59] has revised the mathematical model by a second-order polynomial. Even though the expression is different, it is also a function of oscillating amplitude and reduced wind velocity. It is written as

(21) $$\xi_{a}\left(\hat{y}\right)=a_{1}+a_{2}\;\hat{y}+a_{3}{\;\hat{y}}^{2}$$

or

(22) $$\xi_{a}\left(\sigma_{y}\right)=a_{1}+\sqrt{2}a_{2}\sigma_{y}+2a_{3}\sigma_{y}^{2}$$

where $a_{1},{\;a}_{2}$ and $a_{3}$ are parameters that are functions of reduced wind speed.

Self-excited vibration (galloping) occurs when the total damping of a system is negative. It is in a steady state when the total damping $\xi_{t}=\xi_{a}+\xi_{s}$ becomes zero. The steady state amplitude is determined by

(23) $$\xi_{\mathrm{cr}}+a_{1}+a_{2}\hat{y}+a_{3}{\hat{y}}^{2}=0$$

where $S_{cr}$ is Scruton number and expressed as $S_{cr}=m_{s}\xi_{s}/\left(\rho D^{2}\right)$. $m_{s}$ is the mass ratio.

Solving Equation (23), yields

(24) $$\;\hat{y}=\frac{-a_{2}+\sqrt{a_{2}^{2}-4a_{3}\left(a_{1}+S_{\mathrm{cr}}\right)}}{2a_{3}}$$

**Figure 14:**
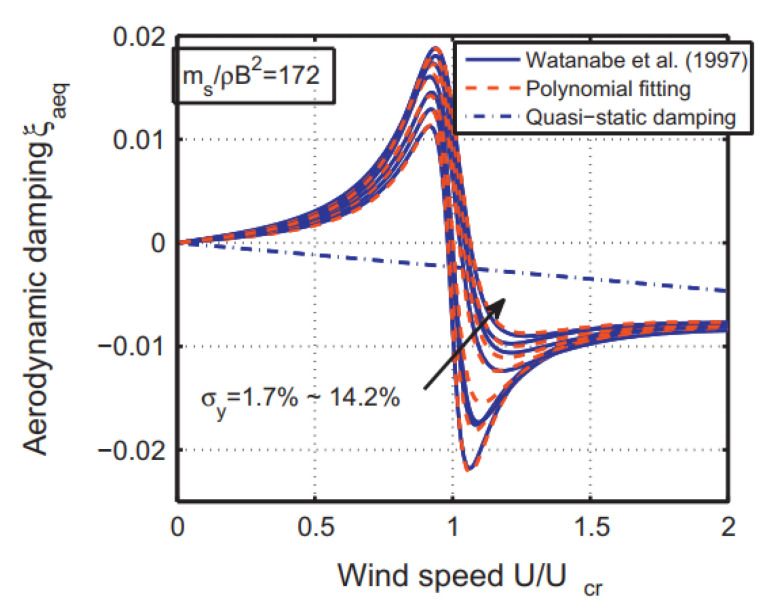
Aerodynamic damping of a test model evaluated by nonlinear mathematical models and the classic quasi-steady theory [59].

By using the developed polynomial, the aerodynamic damping of a test model was evaluated and compared with that estimated by Equation (18) and the quasi-steady theory (Figure 14). The identified aerodynamic damping has been well used for response predictions and fatigue estimations of structures.

Apart from the forced vibration technique, the aerodynamic damping can also be evaluated based on spectral and time series approaches (i.e., auto-regressive (AR) or auto-regressive and moving-averages (ARMA) techniques, half power bandwidth techniques and random decrement techniques (RDT) [62,63,64]). However, these methods have either mathematical limitations or data record problems, and are incorrect in some cases, as reported in previous studies [3,65].

**Figure 15:**
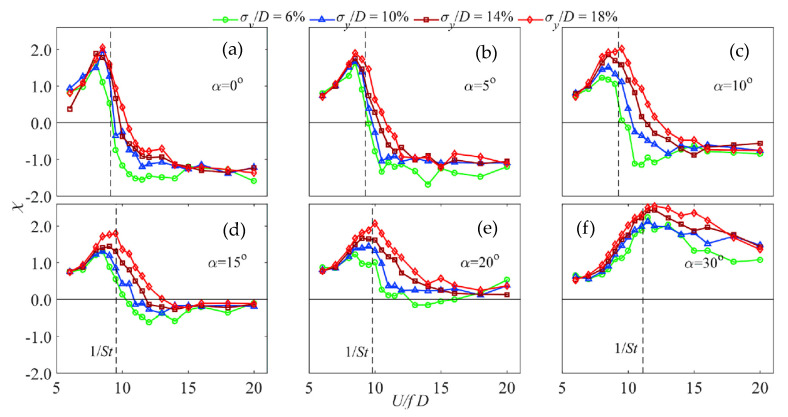
Generalized aerodynamic damping coefficients of forward inclined prisms [33]: (**a**–**f**) with respect to tip amplitude when the inclination angle is equal to 0°, 5°, 10°, 15°, 20° and 30°, respectively.

For forward inclined prisms, the generalized aerodynamic damping coefficients are shown in Figure 15, where $\sigma_{y}/y$ means the normalized amplitude of vibration and inclination and U/fD means reduced wind velocity. α means the inclination angle. It is clear that the generalized aerodynamic damping coefficients reach the peak when the reduced wind speed is 1/St.

### 4.3. Unsteady Self-Excited Force

Chen and Kareem [66] pointed out that aerodynamic forces are commonly separated into static, buffeting and self-excited force components. Among them, the self-excited force component which contains unsteady wind effect is used for predictions of the flutter of deck sections and galloping of prisms. A linear self-excited force model of airfoil and bridge deck has been proposed by Scanlan and Tomo [67]. Due to its simplicity and practicability, it has been widely accepted and used by engineers. Based on the linear model, other self-excited force models with the similar expressions have been proposed [68,69]. Despite the fact that the linear model has proven its utility for many applications, it may not be able to address issues aerodynamic nonlinearities and unsteady effect [66]. Many studies have focused on nonlinear self-excited force models [70,71,72,73,74] that are used for amplitude-dependent flutter derivative identification, high-order flutter force spectrum identification, nonlinear hysteresis analysis and low-speed flutter analysis.

**Figure 16:**
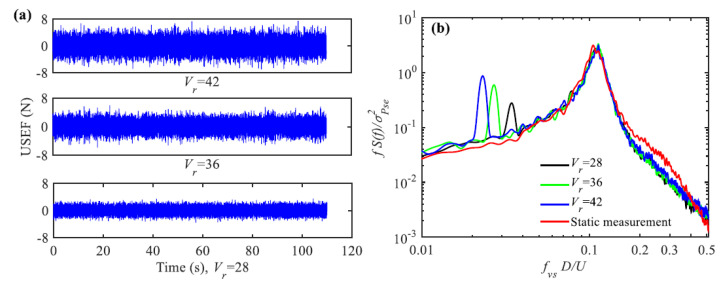
Unsteady aerodynamic forces on the prism: (**a**) In time domain; (**b**) in frequency domain, *f*_vs_ is the frequency of shedding vortices [25].

**Figure 17:**
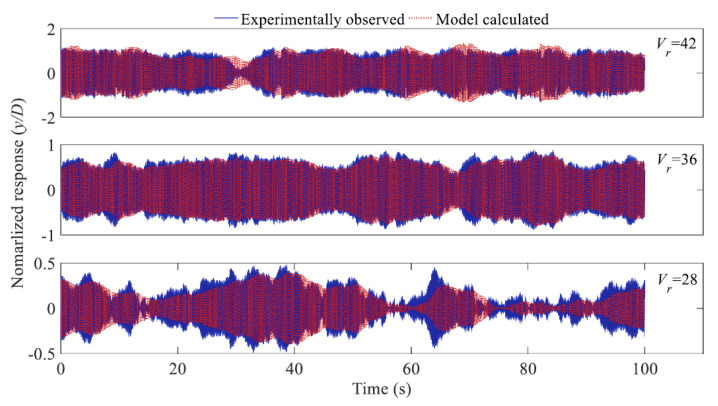
Comparison between the calculated and measured amplitudes of galloping instability [25].

Self-excited galloping force is commonly estimated by a quasi-static method. However, the quasi-static force excludes the effect of structural oscillation and is therefore not applicable to predict galloping responses of structures with respect to unsteady effect. Bouclin and Geoola [75] have proposed a wake-oscillator model which combined the quasi-static force model with the Hartlen-Currie lift model to explain the galloping phenomena of a square cylinder. The classic quasi-static force model was, therefore, improved by introducing the forces induced by the vortex shedding process. Corless and Parkinson [76,77] improved the wake-oscillator model to predict the VIV-galloping combined response of cylinders. Tamura [78] has combined the quasi-static force model with the Birkhoff wake-oscillator model [79] to evaluate the combined effects of VIV and galloping. Mannini et al. [80] have investigated the self-excited force models proposed by Corless and Parkinson, and Tamura through wind tunnel studies on VIV-galloping of rectangular cylinders. They pointed out that though the self-excited force models can substantially reflect the interaction of VIV and galloping, they cannot well predict the combined vibrations. Most recently, Gao and Zhu [41,81] have identified the unsteady galloping force of two-dimensional cylinders by using the aforementioned hybrid aeroelastic-force experimental system (Figure 7). Chen et al. [25] measured the unsteady aerodynamic force and galloping response of a prism from a HAFB wind tunnel test. The unsteady aerodynamic force measured form the hybrid aeroelastic-pressure balance test includes galloping force components and buffeting force components. For the cases of *V*_r_ = 28, 36 and 42, unsteady aerodynamic forces on the prism are shown in Figure 16. For the static measurement case, the only peak was induced by vortex shedding. This means in a static measurement case, the effect of structural motion on aerodynamic force was excluded which will lead to underestimation in the prediction of wind-induced response. The galloping responses of the cylinders predicted by the unsteady galloping force are coincident well with the experimentally observed (Figure 17), suggesting the identified unsteady galloping force is reasonable and reliable.

The identified unsteady galloping force includes three components: an aerodynamic damping component, an aerodynamic stiffness component and a residual force (buffeting force) component. Among them, only the aerodynamic damping or stiffness component inputs or dissipates energy to an oscillating system. Additionally, it has been validated that the effect of the aerodynamic stiffness is slight. On these considerations, a nonlinear mathematical model that is simplified as a fifth order polynomial was established to model the unsteady galloping force. It is expressed as Equation (25).

(25) $$P_{\mathrm{se}}\left(y,\dot{y}\right)=\int_{0}^{H}\frac{1}{2}{\rho U}^{2}D\phi \left(z\right)\mathrm{dz}\left(p_{1}\frac{\dot{y}}{U}+p_{2}\frac{{\dot{y}}^{3}}{U^{3}}+p_{3}\frac{{\dot{y}}^{5}}{U^{5}}\right)+\left(p_{4}y+p_{5}y^{5}+p_{6}y^{6}\right)$$

where $P_{\mathrm{se}}\left(y,\dot{y}\right)$ is unsteady self-excited forces acting on a bluff body; D the height of the test model; ϕ(z) is the mode shape; y is the response of the model; $\dot{y}$ is the velocity of the model; $p_{1},{\;p}_{2},{\;p}_{3},p_{4},p_{5}{\;\mathrm{and}\;p}_{6}$ are the aerodynamic coefficients of unsteady self-excited forces.

**Figure 18:**
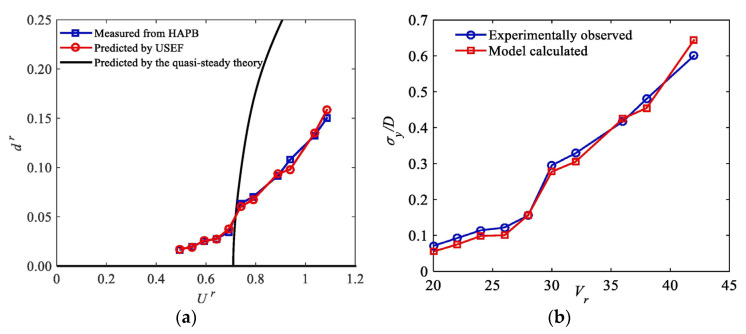
Comparison of predicted amplitudes of oscillation with experimental results and those predicted by the classical quasi-steady theory: (**a**) Galloping response predicted by quasi-steady theory and measured unsteady self-excited force (USEF); (**b**) galloping response calculated by developed model [25].

It was found that the classical quasi-steady theory fails to predict the galloping response of a slender prism, as presented in Figure 18a. By contrast, the galloping response predicted by the developed model is in close agreement with experimental results, as shown in Figure 18b. This suggests that the shortcomings of the classical quasi-steady theory in predicting galloping instabilities of bluff bodies can be well address by the developed model that is established according to unsteady aerodynamic forces measured from the HAPB test.

## 5. Comparison and Perspective

### 5.1. Comparison of Wind Tunnel Test Techniques

As mentioned before, conventional static measurement tests fail to consider the unsteady effect, and the effect of structural vibration is not included. Particularly speaking, the test models used in the HFBB test technique and the SMPSS test technique are static, so the unsteady effect is completely excluded. For bluff bodies undergoing galloping, the quasi-steady theory fails because the unsteady effect on this occasion is very obvious. In this situation, the HFBB test technique and the SMPSS test technique should not be used. For the aeroelastic wind tunnel test, it can experience the fluid–structure interaction like a real building. However, only the wind-induced response of the prism can be observed, and the distributed pressure on the structure cannot be obtained. So aeroelastic characteristics of bluff bodies can be analyzed according to the data obtained from aeroelastic wind tunnel test. Aerodynamic characteristics cannot be received. To overcome the above shortcomings, the forced vibration test technique and HAFB test technique are proposed. They can be considered as the combination of SMPSS and aeroelastic test. The forced vibration test technique is widely performed to obtain local pressure and response of the test model. The model can oscillate at a certain frequency in the crosswind direction due to the motor fixed under the plate. But the technique only considers the one-way coupling effect and the structural motion is not like the real one. To some extent, forced vibration test can be used to obtain aerodynamic force acting on bluff bodies including aerodynamic damping force and aerodynamic stiffness force. But response of prisms in the real world cannot be predicted according to this technique. Due to the disadvantages of conventional wind tunnel test techniques and forced vibration test, HAFB test is developed with the purpose of improving the accuracy of prediction and obtaining more information. The detailed comparison of each test technique is shown in Table 1.

### 5.2. Analytical Scheme of Conventional and Advanced Wind Tunnel Test Techniques

**Figure 19:**
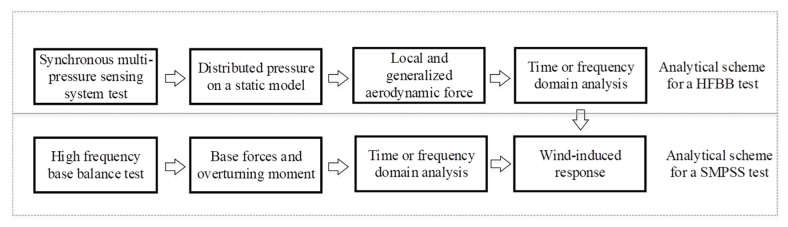
Analytical scheme for a high frequency base balance test or static synchronous multi-pressure sensing system test.

**Figure 20:**
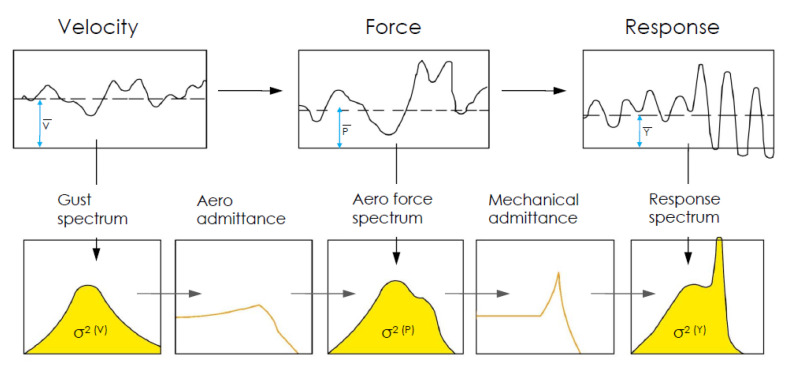
Elements of the statistical approach to gust loading [82].

HFBB is widely applied to predict the wind-induced base shear and moment of bluff bodies. In a HFBB wind tunnel test, generalized wind force and base force can be obtained, which can be used for calculating wind force spectra and wind-induced response (Figure 19) according to a statistical approach (Figure 20).

Despite the effectiveness of the HFBB test technique in analyzing aerodynamics in bluff bodies, it cannot obtain the point wind pressure, cross-correlation coefficient and coherence coefficient of wind pressure on the surface of a test model. SMPSS wind tunnel test can observe wind pressures on a static model synchronously. Integrating the observed wind pressure along the height of a test model, the local and generalized aerodynamic forces can be obtained. Wind-induced responses of the test model are, therefore, calculated according to the analytical scheme (Figure 19) using a statistical approach (Figure 20).

Wind-induced response of a test model cannot be directly obtained from a HFBB or SMPSS wind tunnel test. Aeroelastic test is a straightforward way to measure wind-induced displacement response, torsion angle and acceleration. However, it cannot synchronously observe wind pressures or wind forces acting on a test model.

**Figure 21:**
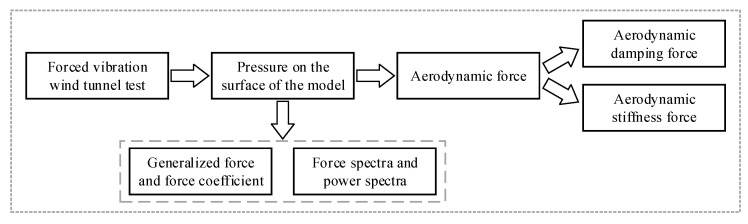
Calculation procedure of forced vibration wind tunnel test.

In a forced vibration wind tunnel test, the unsteady effect of wind acting on a prism can be considered. A certain amplitude and frequency were given to determine the motion of the model. Pressure on the surface of the model was obtained through pressure taps. Then the generalized force and force coefficient, force spectra and power spectra were calculated. Besides, aerodynamic force, aerodynamic damping force and aerodynamic stiffness force can be identified. The calculation procedure for results obtained from a forced vibration wind tunnel test was shown in Figure 21.

**Figure 22:**
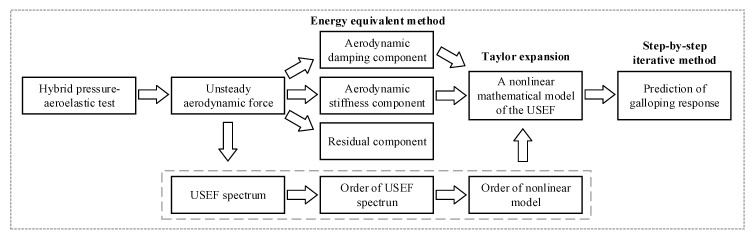
Scheme for modeling the unsteady self-excited force and predicting the galloping response of a bluff body.

In a hybrid aeroelastic-pressure/force balance test, the unsteady effect was fully considered. Unsteady aerodynamic force was divided into three parts including aerodynamic damping component, aerodynamic stiffness component and residual component. According to the first two parts of the three components, a nonlinear mathematical model of the unsteady self-excited force can be deduced, while the order of the nonlinear model was obtained from order of unsteady self-excited force spectrum. As a result, the galloping response of a prism can be predicted. In general, the scheme to model the unsteady self-excited force on a bluff body is summarized in Figure 22.

## 6. Concluding Remarks and Recommendations

In this paper, conventional wind tunnel test techniques (including HFBB, SMPSS and aeroelastic test techniques) and advanced wind tunnel test techniques (including forced vibration test techniques and hybrid aerodynamic-pressure/force balance test techniques) were comprehensively overviewed. The main conclusions and recommendations are summarized as follows.

(1) The HFBB and SMPSS techniques neglect the unsteady effect, which has been proved to have a significant influence on the predicted wind-induced response. The response of the bluff bodies cannot be obtained directly from these two techniques.
(2) The aeroelastic test can only obtain the wind-induced response of a bluff body, while the wind force acting on the model cannot be obtained.
(3) Unsteady aerodynamic forces obtained from a forced vibration wind tunnel test takes part of the unsteady effect into consideration, because a test model is forced to oscillate and it only considers the effect from an oscillating model to its surrounding flow and cannot consider the feedback. As a result, unsteady aerodynamic forces on a forced oscillation model may have considerable differences with those on an aeroelastic model or a structure in the real world.
(4) The hybrid aeroelastic-pressure/force balance test techniques are effective in measuring unsteady aerodynamic forces on bluff bodies during aeroelastic oscillations. The obtained forces can well include the ‘unsteady’ effect and can be used to address the shortcomings of classical theories (e.g., the classical quasi-steady theory) in predicting wind-induced responses of bluff bodies.
(5) The HAPB/HAFB test techniques are highly recommended to investigate the unsteady aerodynamics and aeroelasticity of bluff bodies.

## Figures and Tables

**Table 1:** Comparisons of each wind tunnel test technique.

Test Techniques	Characteristics of Test Model	Measured Content	Disadvantages
High frequency base balance test	Static	Base shear force and base overturning moment	No distributed pressure and unsteady effect
Static synchronous multi-pressure sensing system test	Static	Local pressure	No unsteady effect
Aeroelastic test	Free vibration	Response	No distributed pressure
Forced vibration test	Forced vibration	Local pressure and response	No two-way coupling effect
Hybrid aeroelastic-force balance test	Free vibration	Local pressure and response	−
